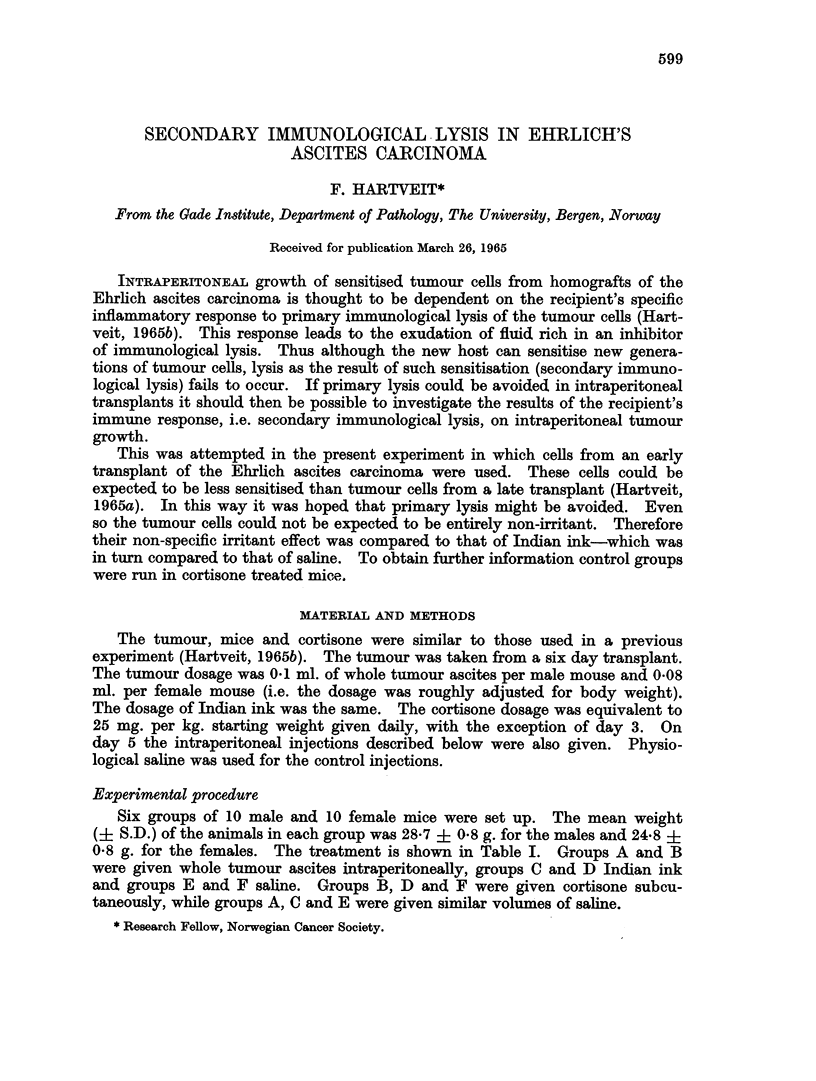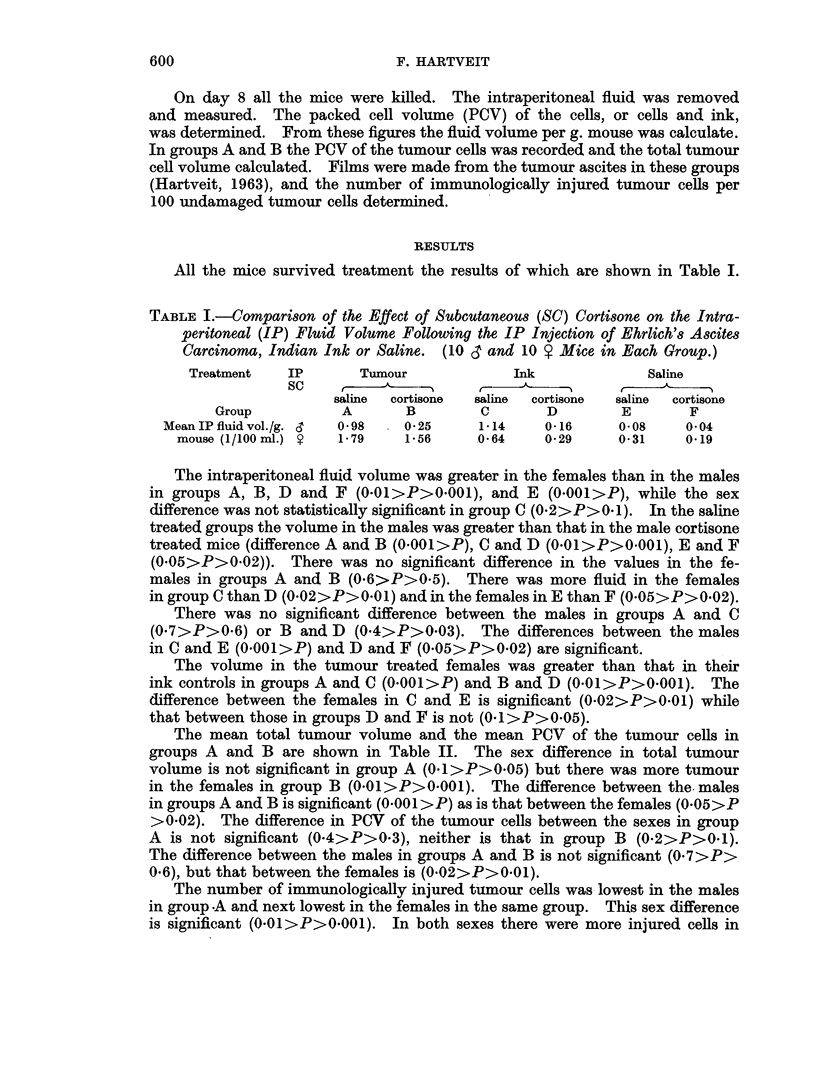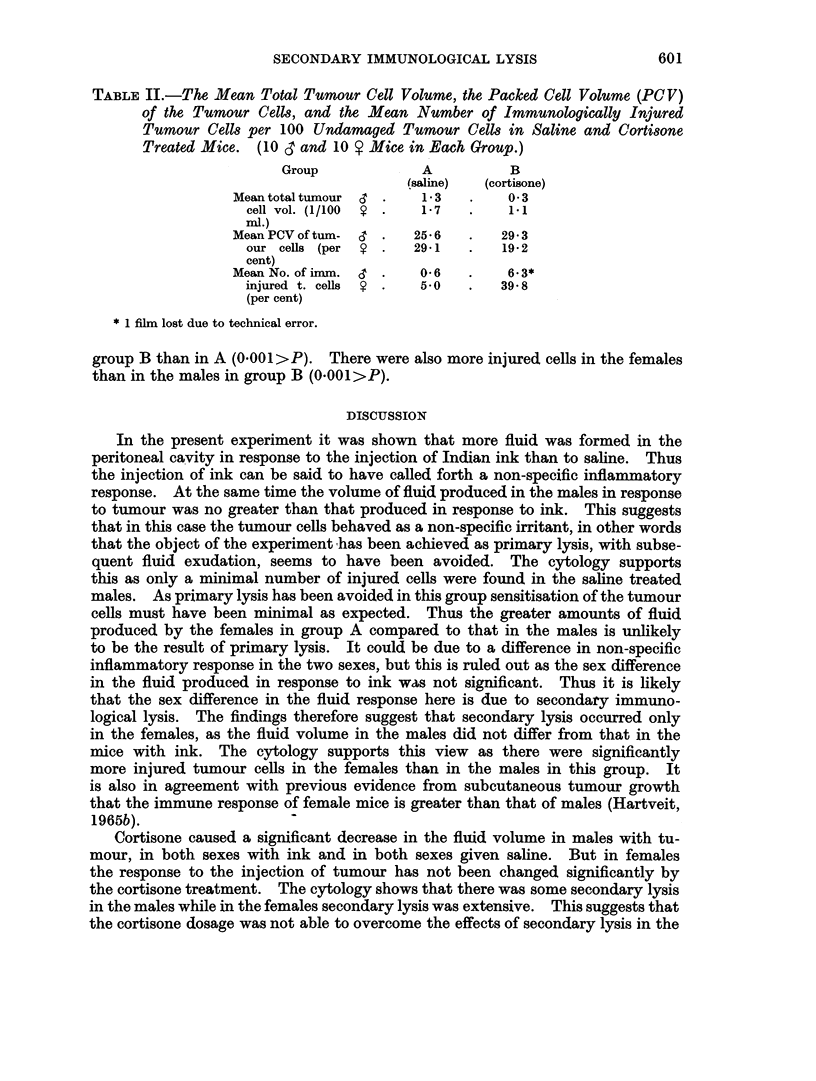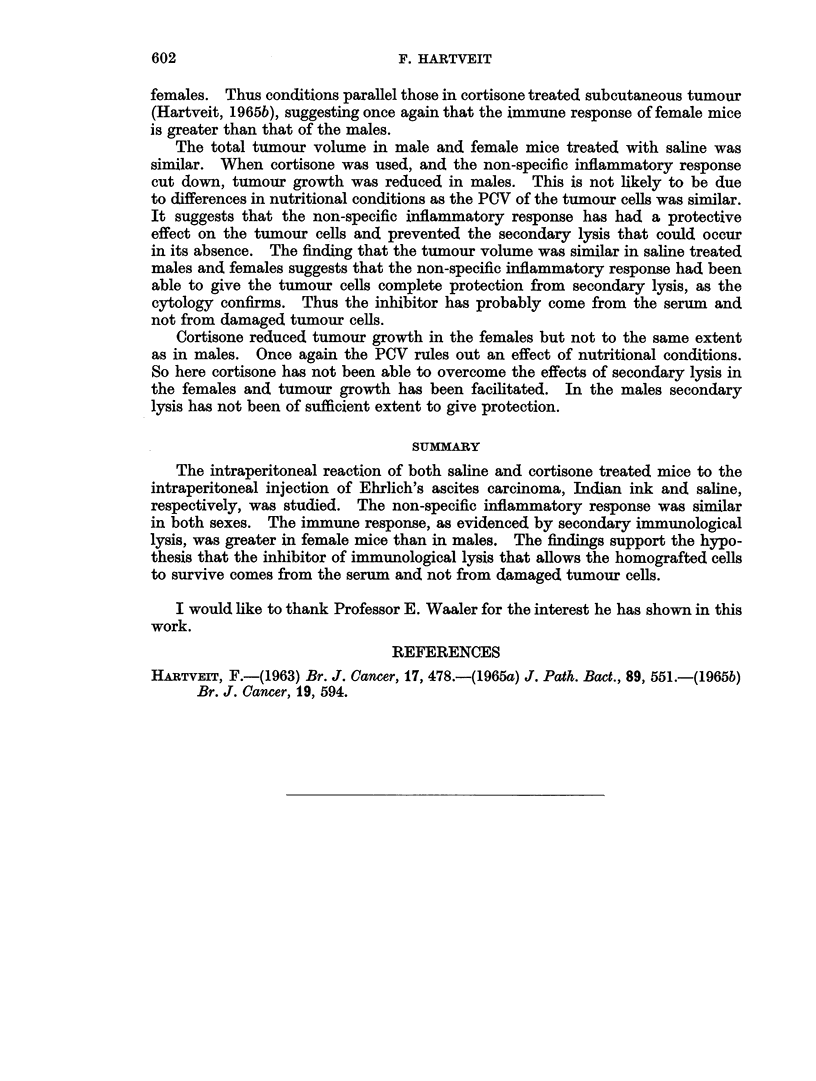# Secondary immunological lysis in Ehrlich's ascites carcinoma.

**DOI:** 10.1038/bjc.1965.75

**Published:** 1965-09

**Authors:** F. Hartveit


### SECONDARY IMMUNOLOGICAL LYSIS IN EHRLICH'S ASCITES CARCINOMA

F. HARTVEIT*

*From the Gade Institute, Department of Pathology, The University, Bergen, Norway*

Received for publication March 26, 1965

INTRAPERITONEAL growth of sensitised tumour cells from homografts of the Ehrlich ascites carcinoma is thought to be dependent on the recipient's specific inflammatory response to primary immunological lysis of the tumour cells (Hartveit, 1965*b*). This response leads to the exudation of fluid rich in an inhibitor of immunological lysis. Thus although the new host can sensitise new generations of tumour cells, lysis as the result of such sensitisation (secondary immunological lysis) fails to occur. If primary lysis could be avoided in intraperitoneal transplants it should then be possible to investigate the results of the recipient's immune response, i.e. secondary immunological lysis, on intraperitoneal tumour growth.

This was attempted in the present experiment in which cells from an early transplant of the Ehrlich ascites carcinoma were used. These cells could be expected to be less sensitised than tumour cells from a late transplant (Hartveit, 1965*a*). In this way it was hoped that primary lysis might be avoided. Even so the tumour cells could not be expected to be entirely non-irritant. Therefore their non-specific irritant effect was compared to that of Indian ink—which was in turn compared to that of saline. To obtain further information control groups were run in cortisone treated mice.

### MATERIAL AND METHODS

The tumour, mice and cortisone were similar to those used in a previous experiment (Hartveit, 1965*b*). The tumour was taken from a six day transplant. The tumour dosage was 0·1 ml. of whole tumour ascites per male mouse and 0·08 ml. per female mouse (i.e. the dosage was roughly adjusted for body weight). The dosage of Indian ink was the same. The cortisone dosage was equivalent to 25 mg. per kg. starting weight given daily, with the exception of day 3. On day 5 the intraperitoneal injections described below were also given. Physiological saline was used for the control injections.

### *Experimental procedure*

Six groups of 10 male and 10 female mice were set up. The mean weight ($\pm$ S.D.) of the animals in each group was 28·7 $\pm$ 0·8 g. for the males and 24·8 $\pm$ 0·8 g. for the females. The treatment is shown in Table I. Groups A and B were given whole tumour ascites intraperitoneally, groups C and D Indian ink and groups E and F saline. Groups B, D and F were given cortisone subcutaneously, while groups A, C and E were given similar volumes of saline.

* Research Fellow, Norwegian Cancer Society.

On day 8 all the mice were killed. The intraperitoneal fluid was removed and measured. The packed cell volume (PCV) of the cells, or cells and ink, was determined. From these figures the fluid volume per g. mouse was calculate. In groups A and B the PCV of the tumour cells was recorded and the total tumour cell volume calculated. Films were made from the tumour ascites in these groups (Hartveit, 1963), and the number of immunologically injured tumour cells per 100 undamaged tumour cells determined.

### RESULTS

All the mice survived treatment the results of which are shown in Table I.

TABLE I.—*Comparison of the Effect of Subcutaneous (SC) Cortisone on the Intraperitoneal (IP) Fluid Volume Following the IP Injection of Ehrlich's Ascites Carcinoma, Indian Ink or Saline.* (10 ♂ *and* 10 ♀ *Mice in Each Group.*)

| Treatment | IP | Tumour | | Ink | | Saline | |
|---|---|---|---|---|---|---|---|
| | SC | saline | cortisone | saline | cortisone | saline | cortisone |
| Group | | A | B | C | D | E | F |
| Mean IP fluid vol./g. | ♂ | 0·98 | 0·25 | 1·14 | 0·16 | 0·08 | 0·04 |
| mouse (1/100 ml.) | ♀ | 1·79 | 1·56 | 0·64 | 0·29 | 0·31 | 0·19 |

The intraperitoneal fluid volume was greater in the females than in the males in groups A, B, D and F (0·01>$P$>0·001), and E (0·001>$P$), while the sex difference was not statistically significant in group C (0·2>$P$>0·1). In the saline treated groups the volume in the males was greater than that in the male cortisone treated mice (difference A and B (0·001>$P$), C and D (0·01>$P$>0·001), E and F (0·05>$P$>0·02)). There was no significant difference in the values in the females in groups A and B (0·6>$P$>0·5). There was more fluid in the females in group C than D (0·02>$P$>0·01) and in the females in E than F (0·05>$P$>0·02).

There was no significant difference between the males in groups A and C (0·7>$P$>0·6) or B and D (0·4>$P$>0·03). The differences between the males in C and E (0·001>$P$) and D and F (0·05>$P$>0·02) are significant.

The volume in the tumour treated females was greater than that in their ink controls in groups A and C (0·001>$P$) and B and D (0·01>$P$>0·001). The difference between the females in C and E is significant (0·02>$P$>0·01) while that between those in groups D and F is not (0·1>$P$>0·05).

The mean total tumour volume and the mean PCV of the tumour cells in groups A and B are shown in Table II. The sex difference in total tumour volume is not significant in group A (0·1>$P$>0·05) but there was more tumour in the females in group B (0·01>$P$>0·001). The difference between the males in groups A and B is significant (0·001>$P$) as is that between the females (0·05>$P$>0·02). The difference in PCV of the tumour cells between the sexes in group A is not significant (0·4>$P$>0·3), neither is that in group B (0·2>$P$>0·1). The difference between the males in groups A and B is not significant (0·7>$P$>0·6), but that between the females is (0·02>$P$>0·01).

The number of immunologically injured tumour cells was lowest in the males in group A and next lowest in the females in the same group. This sex difference is significant (0·01>$P$>0·001). In both sexes there were more injured cells in

TABLE II.—*The Mean Total Tumour Cell Volume, the Packed Cell Volume (PCV) of the Tumour Cells, and the Mean Number of Immunologically Injured Tumour Cells per* 100 *Undamaged Tumour Cells in Saline and Cortisone Treated Mice.* (10 ♂ *and* 10 ♀ *Mice in Each Group.*)

| Group | | A (saline) | B (cortisone) |
|---|---|---|---|
| Mean total tumour cell vol. (1/100 ml.) | ♂ | 1·3 | 0·3 |
| | ♀ | 1·7 | 1·1 |
| Mean PCV of tumour cells (per cent) | ♂ | 25·6 | 29·3 |
| | ♀ | 29·1 | 19·2 |
| Mean No. of imm. injured t. cells (per cent) | ♂ | 0·6 | 6·3* |
| | ♀ | 5·0 | 39·8 |

* 1 film lost due to technical error.

group B than in A ($0{\cdot}001 > P$). There were also more injured cells in the females than in the males in group B ($0{\cdot}001 > P$).

### DISCUSSION

In the present experiment it was shown that more fluid was formed in the peritoneal cavity in response to the injection of Indian ink than to saline. Thus the injection of ink can be said to have called forth a non-specific inflammatory response. At the same time the volume of fluid produced in the males in response to tumour was no greater than that produced in response to ink. This suggests that in this case the tumour cells behaved as a non-specific irritant, in other words that the object of the experiment has been achieved as primary lysis, with subsequent fluid exudation, seems to have been avoided. The cytology supports this as only a minimal number of injured cells were found in the saline treated males. As primary lysis has been avoided in this group sensitisation of the tumour cells must have been minimal as expected. Thus the greater amounts of fluid produced by the females in group A compared to that in the males is unlikely to be the result of primary lysis. It could be due to a difference in non-specific inflammatory response in the two sexes, but this is ruled out as the sex difference in the fluid produced in response to ink was not significant. Thus it is likely that the sex difference in the fluid response here is due to secondary immunological lysis. The findings therefore suggest that secondary lysis occurred only in the females, as the fluid volume in the males did not differ from that in the mice with ink. The cytology supports this view as there were significantly more injured tumour cells in the females than in the males in this group. It is also in agreement with previous evidence from subcutaneous tumour growth that the immune response of female mice is greater than that of males (Hartveit, 1965*b*).

Cortisone caused a significant decrease in the fluid volume in males with tumour, in both sexes with ink and in both sexes given saline. But in females the response to the injection of tumour has not been changed significantly by the cortisone treatment. The cytology shows that there was some secondary lysis in the males while in the females secondary lysis was extensive. This suggests that the cortisone dosage was not able to overcome the effects of secondary lysis in the females. Thus conditions parallel those in cortisone treated subcutaneous tumour (Hartveit, 1965*b*), suggesting once again that the immune response of female mice is greater than that of the males.

The total tumour volume in male and female mice treated with saline was similar. When cortisone was used, and the non-specific inflammatory response cut down, tumour growth was reduced in males. This is not likely to be due to differences in nutritional conditions as the PCV of the tumour cells was similar. It suggests that the non-specific inflammatory response has had a protective effect on the tumour cells and prevented the secondary lysis that could occur in its absence. The finding that the tumour volume was similar in saline treated males and females suggests that the non-specific inflammatory response had been able to give the tumour cells complete protection from secondary lysis, as the cytology confirms. Thus the inhibitor has probably come from the serum and not from damaged tumour cells.

Cortisone reduced tumour growth in the females but not to the same extent as in males. Once again the PCV rules out an effect of nutritional conditions. So here cortisone has not been able to overcome the effects of secondary lysis in the females and tumour growth has been facilitated. In the males secondary lysis has not been of sufficient extent to give protection.

### SUMMARY

The intraperitoneal reaction of both saline and cortisone treated mice to the intraperitoneal injection of Ehrlich's ascites carcinoma, Indian ink and saline, respectively, was studied. The non-specific inflammatory response was similar in both sexes. The immune response, as evidenced by secondary immunological lysis, was greater in female mice than in males. The findings support the hypothesis that the inhibitor of immunological lysis that allows the homografted cells to survive comes from the serum and not from damaged tumour cells.

I would like to thank Professor E. Waaler for the interest he has shown in this work.

### REFERENCES

HARTVEIT, F.—(1963) *Br. J. Cancer*, **17**, 478.—(1965*a*) *J. Path. Bact.*, **89**, 551.—(1965*b*) *Br. J. Cancer*, **19**, 594.